# Social Exclusion Index-for Health Surveys (SEI-HS): a prospective nationwide study to extend and validate a multidimensional social exclusion questionnaire

**DOI:** 10.1186/s12889-017-4175-1

**Published:** 2017-03-14

**Authors:** Addi P. L. van Bergen, Stella J. M. Hoff, Hanneke Schreurs, Annelies van Loon, Albert M. van Hemert

**Affiliations:** 1 Department of Public Health, Municipality of Utrecht, Utrecht, Netherlands; 2 Department of Labour and Public Services, The Netherlands Institute of Social Research|SCP, The Hague, Netherlands; 3 Department of Epidemiology and Health Promotion, Public Health Service of Amsterdam, Amsterdam, Netherlands; 4 Department of Psychiatry, Leiden University Medical Centre, Leiden, Netherlands

**Keywords:** Social exclusion, Public health monitoring, Health inequalities, OVERALS, Non-linear canonical correlation analysis, Embedded measure

## Abstract

**Background:**

Social exclusion (SE) refers to the inability of certain groups or individuals to fully participate in society. SE is associated with socioeconomic inequalities in health, and its measurement in routine public health monitoring is considered key to designing effective health policies. In an earlier retrospective analysis we demonstrated that in all four major Dutch cities, SE could largely be measured with existing local public health monitoring data. The current prospective study is aimed at constructing and validating an extended national measure for SE that optimally employs available items.

**Methods:**

In 2012, a stratified general population sample of 258,928 Dutch adults completed a version of the Netherlands Public Health Monitor (PHM) questionnaire in which 9 items were added covering aspects of SE that were found to be missing in our previous research. Items were derived from the SCP social exclusion index, a well-constructed 15-item instrument developed by the Netherlands Institute for Social Research (SCP). The dataset was randomly divided into a development sample (*N* =129,464) and a validation sample (*N* = 129,464). Canonical correlation analysis was conducted in the development sample. The psychometric properties were studied and compared with those of the original SCP index. All analyses were then replicated in the validation sample.

**Results:**

The analysis yielded a four dimensional index, the Social Exclusion Index for Health Surveys (SEI-HS), containing 8 SCP items and 9 PHM items. The four dimensions: “lack of social participation”, “material deprivation”, “lack of normative integration” and “inadequate access to basic social rights”, were each measured with 3 to 6 items. The SEI-HS showed adequate internal consistency for both the general index and for two of four dimension scales. The internal structure and construct validity of the SEI-HS were satisfactory and similar to the original SCP index. Replication of the SEI-HS in the validation sample confirmed its generalisability.

**Conclusion:**

This study demonstrates that the SEI-HS offers epidemiologists and public health researchers a uniform, reliable, valid and efficient means of assessing social exclusion and its underlying dimensions. The study also provides valuable insights in how to develop embedded measures for public health surveillance.

**Electronic supplementary material:**

The online version of this article (doi:10.1186/s12889-017-4175-1) contains supplementary material, which is available to authorized users.

## Background

Socioeconomic inequalities in health are one of the major challenges in the field of public health today. Social, material, cultural and political conditions shape our lives and our behaviours and thereby influence our health [1]. Social exclusion (SE) is understood to be one of the drivers of inequalities in health [1–3]. SE refers to the inability of certain groups or individuals to participate fully in society due to personal and societal factors. SE is a multidimensional concept, involving cumulative disadvantages in the social, economic, cultural and political domains [4–7]. The concept of SE is regarded as a promising entry for addressing health inequalities [6–8]. Not only do the circumstances associated with SE such as poverty, poor housing, few social contacts and reduced access to care, have a negative impact on health, also the actual experience of exclusion may impact negatively on health status via psychosocial stress mechanisms [2, 7, 9, 10]. Poor physical and mental health, in turn, can be a barrier to social and economic participation [11].

To address health inequalities at local or national level, it is important to gain insight into the prevalence and nature of SE and its relationship with health. However, a generally accepted measure of SE does not yet exist in public health research [6, 8, 12–15]. Health research typically focuses on a single dimension of SE, such as poverty, labour market exclusion or access to services [6, 8, 16]. Other limitations include the lack of theoretical grounding [16–18], conceptual justification for indicator choice and overall measurement validation [6, 8]. SE measures that have been validated are, to our knowledge, not particularly suited for use in public health surveys. These measures were developed for use in specific target populations instead of the general population [19–29], are too lengthy for use in population surveys [20, 30], do not allow for self-report [26–28] or measure health as a constituent part of SE [15, 31].

The lack of a suitable measure for SE prompted us in a previous study to develop our own instrument using existing routine public health survey data of the four major cities in the Netherlands [32]. As the gold standard we used the social exclusion index of the Netherlands Institute for Social Research|SCP (SCP) [33, 34], which was developed for use in social and economic policy research. This index does not suffer from the above limitations: it is multidimensional, theoretically sound, thoroughly validated, designed for use in the general population, brief, with only 15 items, suitable for self-report, not including a health domain and providing an overall index [33, 34].

The SCP index is the result of a decade of research and reflection [5, 35]. It is rooted in two main theoretical conceptualisations of SE: the French scientific tradition, in which SE refers to the socio-cultural aspects of people’s lives, the extent to which people are integrated into society and their connection with others; and the Anglo-Saxon line, in which SE is associated with structural-economic aspects of people’s lives, with relative deprivation and unequal access to income, basic goods, public services and citizen rights ([5, 33], cf. eg. [17, 36–39]). The SCP index is composed of two dimensions that concur with the French tradition i.e. (lack of) Social Participation (regarding social isolation and limited participation in social networks) and (lack of) Normative Integration (referring to non-compliance with core values of society); and two dimensions that concur with the Anglo-Saxon line i.e. Material Deprivation (deficits that people experience as shown by debts and the absence of certain basic goods and services) and (inadequate access to basic) Social Rights (referring to the people’s inability to exercise their citizens’ rights).

The SCP Index, however, proved ill-suited for use in routine public health monitoring due to a substantial overlap with current topics, such as loneliness, social capital, financial situation and housing, and lack of space for 15 additional items. Our previous study [32] showed that in all four cities, the above described multidimensional concept of SE could be validly approximated with existing data from public health questionnaires. From each questionnaire we had selected the items that corresponded to those of the SCP-instrument and entered these into a nonlinear canonical correlation analysis. The internal consistency of the resulting indices was adequate to good, and so were the internal structure, generalisability and construct validity. The content validity however, was only moderate. The dimension scales for Material Deprivation and Social Rights did not cover the full width of the theoretical constructs. The Material Deprivation scales missed items on lack of basic goods and services such as club membership and heating one’s home. The Social Rights scales missed an item on the actual lack of access to healthcare. Such items were not available in the health questionnaires of the four cities. One of the SE dimensions, i.e. the dimension Normative Integration, could not be measured at all due to lack of appropriate items in the survey questionnaires. Another limitation of our study was that replication of the indices was confined to urban areas only.

In the current prospective study we addressed these limitations by 1) extending the study to the national level and harmonizing with the Netherlands Public Health Monitor and 2) adding extra items to enhance content validity. Our ultimate goal is to develop a nationally validated and standardised measure to monitor SE in routine public health surveys among adults, that optimally employs available survey items.

In the Netherlands, routine public health monitoring is carried out by 28 Community Health Services, in cooperation with Statistics Netherlands (CBS) and the Netherlands National Institute for Public Health and the Environment. Every four years, health questionnaires are distributed to a large sample of the Dutch adult population. The monitoring forms part of the health status assessment stage of the Dutch four year preventive care cycle, on the basis of which specific objectives for and the implementation of national and local health policies are defined, implemented and adjusted [40, 41]. Besides mandatory nationwide questions, the health questionnaires also contain optional questions that address local health policy priorities. Community Health Services are obliged to use standard questions developed within the framework of the Netherlands Public Health Monitor (PHM). Only when PHM standard questions are unavailable about a particular subject, can Community Health Services employ other, local, questions [41, 42]. In our effort to construct a national measure for SE, we aimed at making maximum use of the available PHM standard questions, and using supplementary items from the SCP index only where the PHM fell short. In this paper we describe the construction and validation of this embedded measure for SE, the Social Exclusion Index for Health Surveys (SEI-HS).

## Methods

### Data source and participants

This survey study was conducted fall 2012 by 19 of the 28 Dutch Community Health Services who were involved in the implementation of the PHM. These 19 Community Health Service regions cover 71% of the Dutch population. In each Community Health Service region a sample was drawn from the non-institutionalised population aged 19 years and older (as of September 1, 2012), stratified by municipality, neighbourhood and age category (19–64 years and 65 years and older). In total, the 19 samples contained 566,521 persons.

Selected persons received an announcement letter by mail, followed one week later by a questionnaire. The questionnaires could be filled out in writing or online. Non-responders received at least one written reminder. The four largest cities, having a higher proportion of hard to reach groups, made additional efforts such as home visits after the second written reminder, providing translated questionnaires (Turkish, English and Arabic) and offering personal assistance in completing the questionnaire if needed. Questionnaires were excluded if two third or more of the SE questions were not answered or in the case of lacking information on at least two thirds of the core questions. According to the national protocol, core questions include a.o. educational level, employment status, body weight and smoking. The net response rate was 45.7% (258,928 respondents).

Weighting was used to correct for selective non-response and unequal selection probabilities caused by the stratified sampling design. Adjustment weights were calculated for the national sample, based on a linear model with auxiliary variables Community Health Service region (28 categories), gender (2), age (13), marital status (4), degree of urbanisation (5), household size (5), ethnicity (3), income (5) and municipality (391), and their interaction terms [43]. We adjusted these weights in accordance with the sample composition of our study.

### Item selection

In our previous research [32] we identified with nonlinear canonical correlation analysis 16 PHM items from a pool of 62 potential items, measuring various aspects of the four dimensions of SE (Table 1 column 1). Eight of these 16 items are part of the mandatory national questionnaire (PHM1 to PHM7 and PHM9) and are included routinely in the health surveys. The other eight PHM items are optional, meaning that cities could choose not to include these items. After comparison with the SCP index, five of these eight items were considered redundant and were not included in the health surveys. The three remaining optional PHM items were PHM8, PHM10 and PHM14 (Table 1 column 1). From the SCP social exclusion index nine items were added to the surveys to enhance the content validity of the SEI-HS (Table 1 column 2). These items were selected in previous research from an item pool of 232 items covering the broad spectrum of SE [34]. Four SCP items (SCP12 to SCP15) were added to measure Normative Integration, four items (SCP5 to SCP8) to measure Material Deprivation and one item (SCP11) on not receiving medical or dental treatment was added in the dimension Social Rights. In total, 20 items were available for the construction of the SEI-HS.

**Table 1:** Summary of items available for the construction of the SEI-HS, by dimension and source

Items Netherlands Pubic Health Monitor (PHM) identified in prior research	Items SCP index ^&^	Items in the routine public health survey 2012	Excluded in OVERALS analysis
Dimension 1: Limited social participation			
*PHM1.I experience a general sense of emptiness ^#a^	SCP1. I feel cut off from other people	PHM1. I experience a general sense of emptiness ^#^	
*PHM2. There is always someone I can talk to about my day-to-day problems ^#a^	SCP 2. There are people with whom I can have a good conversation	PHM2. There is always someone I can talk to about my day-to-day problems ^#^	
*PHM3. There are plenty of people I can lean on when I have problems ^#a^		PHM3. There are plenty of people I can lean on when I have problems ^#^	
*PHM4. I miss the pleasure of the company of others ^#a^		PHM4. I miss the pleasure of the company of others ^#^	
*PHM5. I often feel rejected ^#a^		PHM5. I often feel rejected ^#^	
*PHM6. I miss having people around ^#a^		PHM6. I miss having people around ^#^	Yes
*PHM7: There are enough people I feel close to ^#a^		PHM7: There are enough people I feel close to ^#^	Yes
	SCP 3. There are people who genuinely understand me		
*PHM8. Little contact with neighbours and people in the street ^$^	SCP 4. I have contact with neighbours	PHM8. Little contact with neighbours and people in the street ^$^	
Dimension 2: Material deprivation			
*PHM9. Had difficulty past year getting by on the household income ^#^		PHM9. Had difficulty past year getting by on the household income ^#^	
	*SCP 5. I have enough money to heat my home	SCP5. I have enough money to heat my home	
	*SCP 6. I have enough money for club memberships	SCP6. I have enough money for club memberships	
	*SCP 7. I have enough money to visit others	SCP7. I have enough money to visit others	
	*SCP 8. I have enough money to meet unexpected expenses	SCP8. I have enough money to meet unexpected expenses	Yes
Dimension 3: Inadequate access to basic social rights			
*PHM10. People in this neighbourhood generally do not get along with each other ^$^	SCP 9. We all get on well in our neighbourhood	PHM10. People in this neighbourhood generally do not get along with each other ^$^	
PHM11. The people in my neighbourhood help each other ^$^			
PHM12. People in this neighbourhood can be trusted ^$^			
PHM13. I prefer not to socialise with people in my neighbourhood ^$^			
*PHM14. Degree of satisfaction with housing ^$^	SCP 10. I am satisfied with the quality of my home	PHM14. Degree of satisfaction with housing ^$^	
PHM15. Feeling unsafe during the day ^$^			
PHM16. Feeling unsafe in the evening and at night ^$^			
	*SCP 11. I didn’t receive a medical or dental treatment	SCP 11. I didn’t receive a medical or dental treatment	
Dimension 4: Lack of normative integration			
	*SCP 12. I give to good causes	SCP 12. I give to good causes	
	*SCP 13. I sometimes do something for my neighbours	SCP 13. I sometimes do something for my neighbours	
	*SCP 14. I put glass items in the bottle bank	SCP 14. I put glass items in the bottle bank	
	*SCP 15. Work is just a way of earning money	SCP 15. Work is just a way of earning money	

*Item included in the routine public health survey 2012

^#^Netherlands Pubic Health Monitor (PHM) mandatory [41]

^$^PHM optional [41]

^&^Vrooman and Hoff [34]

^a^De Jong Gierveld J, Kamphuis FH (1985) The development of a Rasch-type loneliness-scale. Appl Psychol Meas 9: 289–299. doi: 10.1177/014662168500900307

### Construction of the SEI-HS

Nonlinear canonical correlation analysis (OVERALS module in SPSS 19.0) was used to construct a multidimensional index and four underlying dimension scales. OVERALS is a suitable method for the construction of a composite measure as it allows multiple sets of variables (here dimensions of SE), different measurement levels (nominal, ordinal and interval) and distributions [44, 45]. The OVERALS algorithm compares the variable sets to an unknown comprise set that is defined by the object scores [44]. If the correlation between the sets is sufficient, it is assumed that these sets refer to a shared underlying concept [45]. In order to test the generalisability of the extended measure, the dataset was randomly split with SPSS “Select Cases” into a development sample (*N* = 129,464) and a validation sample (*N* = 129,464). All analyses were carried out in the development sample and replicated in the validation sample.

The 20 items were coded in the same direction (low score = little or no exclusion). Based on the OVERALS category quantifications, their measurement level was set as ordinal. Initially all items were entered in the OVERALS analysis, after which items with low component loadings or low weights were removed one by one, until a workable set of items remained. OVERALS weights are considered low at a value of less than 0.100, component loadings at a value of less than 0.300 [44]. Partial cases with maximum three missing values in total and maximum one per dimension were included in the OVERALS analyses.^1^ Since OVERALS does not calculate scores on the subscales, we calculated these by the formula: scale score = Σ transformed item score * item weight. Maximum one missing value was allowed.

### Trichotomisation

As an important application of the SEI-HS in public health policy will be the comparison of SE rates between population groups and monitoring changes over time, we trichotomised both index and scaling scores. The P85 and P95 have been chosen as cut-off points in consultation with Community Health Service epidemiologists. Scores less than or equal to the 85th percentile in the weighted population were labelled “little or no” exclusion, scores greater than the 85th percentile but smaller than or equal to the 95th percentile “some”, and scores greater than the 95th percentile were labelled “moderate to strong” exclusion.

### Measurement properties

The final version of the SEI-HS was evaluated on (1) content validity, (2) internal consistency, (3) structure, (4) construct validity, and (5) generalisability. The analyses were carried out in the development sample and replicated in the validation sample.

#### Content validity

We examined whether all dimensions and aspects of SE of the SCP index were measured by the SEI-HS and compared the distributions of the SEI-HS and the SCP index.

#### Internal consistency

The canonical correlation in OVERALS measures the degree to which the items contribute to the underlying construct of SE. The internal consistency of the index was considered sufficient if the canonical correlation was 0.30 or higher [33, 45]. The internal consistency of the underlying dimension scales was considered sufficient if Cronbach’s alpha was 0.70 or higher [46].

#### Internal structure

We computed the intercorrelations between the subscales and the general index. We expected strong positive correlations between the subscales and the general index (*r* > = 0.60) and sufficient but not strong positive correlations between the subscales (0.20 < = *r* <0.40) [47, 48]. If the correlations between the subscales are sufficient, it is assumed that these scales refer to a shared underlying concept [45]. Additionally, we conducted confirmatory factor analysis in AMOS. We considered a root mean square error of approximation (RMSEA) < 0.05 and upper bound of 90% confidence interval (HI90) < 0.06, Tucker-Lewis index (TLI) ≥ 0.95, comparative fit index (CFI) > 0.90 and Hoelter’s .05 Index ≥ 200 to indicate good model fit [49].

#### Construct validity

We tested a number of hypotheses using linear regression analysis (point biserial correlation). Based on previous research, we expected a positive correlation between the SEI-HS and the following risk factors and correlates: low educational level, non-Western ethnic background, single-parent family with minor children, living alone, low labour market status (and/or recipient of social security or disability benefits), not having paid work, low household income, health problems and living in a deprived neighbourhood. Household income referred to the standardised disposable household income after payment of income tax and social contributions. Low household income corresponded to the lowest income quintile in 2010 (data source: CBS). Health problems included in the study were: fair or poor self-rated health (versus good or very good); being diagnosed with at least one chronic condition; impaired hearing, sight and/or mobility; and high risk for anxiety and depression disorder (score 30 or higher on Kessler psychological distress scale). The significance level for testing was set at 0.001. Construct validity was considered adequate if at least 75% of the hypotheses were confirmed [46].

#### Generalisability

We replicated the construction of the SEI-HS in the validation sample. As suggested in the literature we compared for similarities of the canonical functions [44, 47]. If marked differences are found, the results may be specific to the sample data only and cannot be generalised to the population.

### Statistical analysis

Analyses were carried out using SPSS version 19.0 and SPSS AMOS version 22.0.

## Results

### Participants

Table 2 presents the socio-demographic characteristics of the study sample. The average age in the unweighted sample was 54.8 years and there were slightly more women than men. Compared to the Dutch population as a whole, our study sample was substantially older and included a lower percentage of respondents from (very) highly urbanised areas and from rural areas. Also, men, respondents of non-western ethnic background and respondents with low income were under-represented in the study sample. These differences largely disappeared after weighting for sample coverage and non-response (Table 2).

**Table 2:** Sociodemographic characteristics of the respondents in the study sample (*N* = 258,928) compared to the Dutch population

Characteristics	Study sample	Study sample	Dutch population^a^
	Unweighted	Weighted	
Sex: male (%)	45.2	49.1	49.0
Age (mean, SD)	54.8 (17.7)	48.7 (17.6)	48.8
Ethnic background: non-Western (%)	5.2	10.4	10.2
Educational level: very low (%)^b^	8.7	7.4	7.8
Employment status: Unemployed, recipient of social security or disability benefits. (%)	9.6	10.3	10.6
Income: low (%)^c^	10.5	14.1	14.4
Family situation: living alone (%)	17.3	17.2	17.8
Geographic area: highly urbanised (%)^d^	14.9	20.2	20.2
Geographic area: rural (%)^e^	14.5	10.7	10.7

^a^Data source: Sex, ethnicity and urbanisation: Statistics Netherlands 2012 (statline.cbs.nl); Other data: PHM 2012

^b^No education and primary school

^c^Low income = lowest quintile standardised yearly household income (2010) i.e. below 15.200 Euro. Data obtained from Statistics Netherlands (CBS)

^d^Municipality with area address density > =2500 adresses per km^2^ (2012). Data obtained from Statistics Netherlands

^e^Municipality with area address density <500 adresses per km^2^ (2012). Data obtained from Statistics Netherlands

### Construction of the SEI-HS

**Fig. 1:**
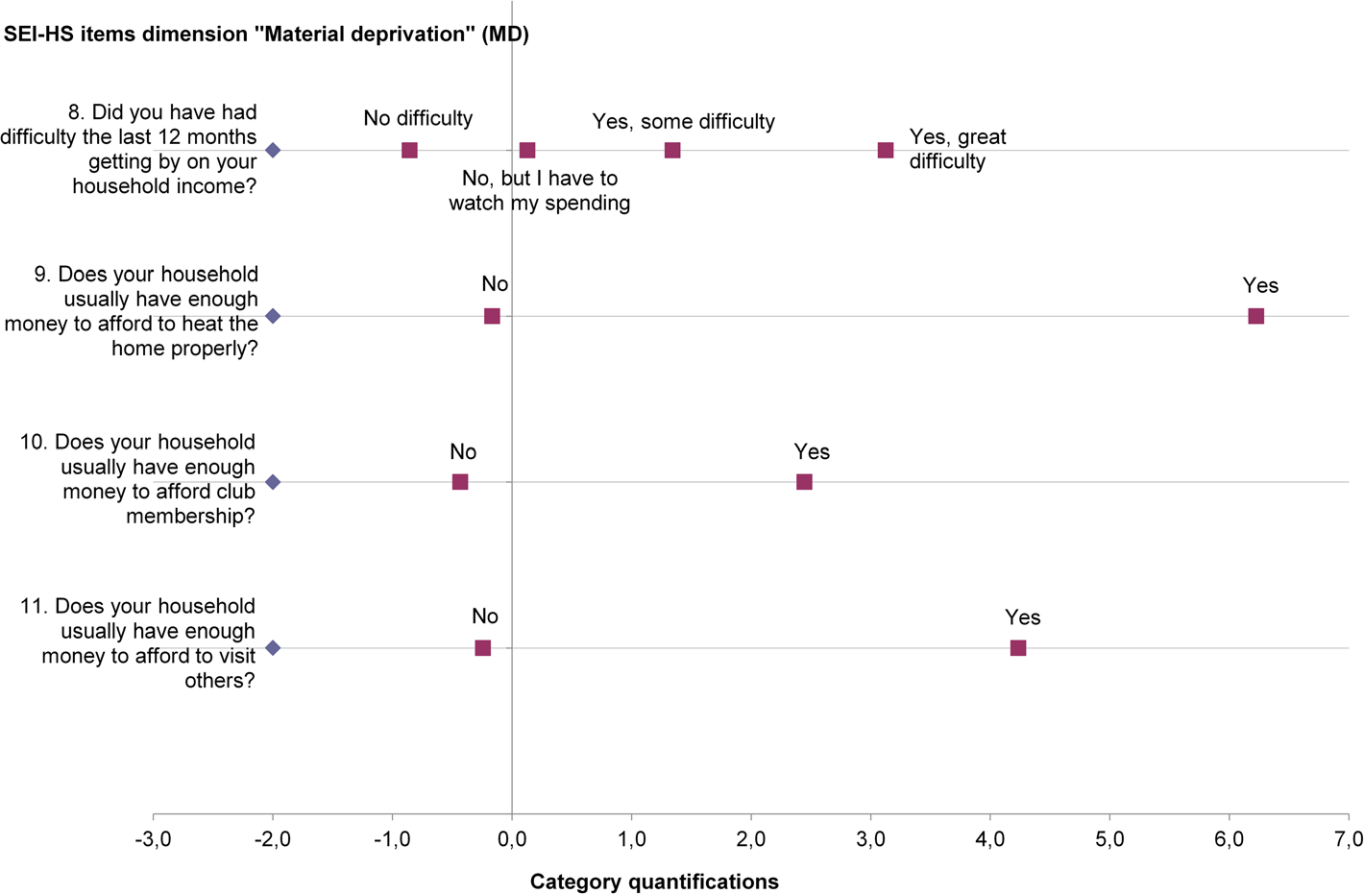
Category quantifications SEI-HS items dimension Material deprivation. Figure 1 shows for each item of the dimension Material Deprivation the relationship between the original category and the quantification resulting from the canonical correlation analysis. Categories indicating little or no social exclusion received the lowest quantifications and categories indicating high levels of social exclusion received the highest values. The category quantifications were used to calculate the Material Deprivation scale score by multiplying them with their item weights (Table 3); and adding up the results

Three of the 20 available items were removed in the final model of the OVERALS analysis (Table 1 last column), while 17 items remained. As shown in Table 3, the dimension (inadequate) Social Participation was measured with 6 items, the dimensions Material Deprivation and (insufficient) Normative Integration were both measured with 4 items, and the dimension (inadequate access to basic) Social Rights with 3 items. Transformed item scores are shown in Fig. 1 (Material Deprivation), Additional file 1 (Social Participation), Additional file 2 (Social Rights) and Additional file 3 (Normative Integration).

**Table 3:** Canonical correlation analysis summary table: SEI-HS (development sample) compared to SCP index

	SEI-HS (*n* = 121,910)				SCP index (*n* = 574)^a^			
	Component loading^b^	Weight^c^	Eigenvalue	α	Component loading^b^	Weight^c^	Eigenvalue	α
Set 1: Limited social participation			0.54	0.75			0.58	0.42
I experience a general sense of emptiness // SCP: I feel cut off from other people	0.49	0.13			0.43	0.27		
There is always someone I can talk to about my day-to-day problems (rev) // SCP: There are people with whom I can have a good conversation	0.42	0.13			0.39	0.18		
Little contact with neighbours and people in the street // SCP: I have contact with neighbours	0.48	0.36			0.52	0.43		
There are plenty of people I can lean on when I have problems (rev)	0.44	0.15						
I miss the pleasure of the company of others	0.51	0.16						
I often feel rejected	0.52	0.23						
There are people who genuinely understand me (rev)					0.51	0.33		
Set 2: Material deprivation			0.49	0.73			0.49	0.57
Had difficulty past year getting by on the household income // SCP: I have enough money to meet unexpected expenses	0.56	0.31			0.53	0.29		
I have enough money to heat my home (rev)	0.36	0.09			0.45	0.32		
I have enough money for club memberships (rev)	0.58	0.30			0.47	0.19		
I have enough money to visit others (rev)	0.52	0.21			0.44	0.24		
Set 3: Inadequate access to basic social rights			0.53	0.30			0.59	0.24
People in this neighbourhood generally do not get along with each other (rev)// SCP: We all get on well in our neighbourhood	0.54	0.43			0.49	0.42		
Degree of satisfaction with housing // SCP: I am satisfied with the quality of my home	0.56	0.45			0.52	0.42		
I didn’t receive a medical or dental treatment	0.27	0.20			0.44	0.38		
Set 4: Lack of normative integration			0.43	0.34			0.47	0.31
I give to good causes (rev)	0.40	0.28			0.38	0.20		
I sometimes do something for my neighbours (rev)	0.41	0.32			0.50	0.43		
I put glass items in the bottle bank (rev)	0.31	0.21			0.38	0.27		
Work is just a way of earning money	0.36	0.29			0.31	0.25		
Eigenvalue index			0.50				0.53	
Canonical correlation^d^			0.33				0.38	

Explanatory note. The table displays component loadings and weights per item, eigenvalue and Cronbach’s alpha per subscale and canonical correlation per index

^a^Vrooman and Hoff [34]

^b,c^Component loadings in OVERALS are similar to factor loadings in a factor analysis. Weights are similar to standardised regression coefficients [44, 45]

^d^The canonical correlation is calculated with the formula: r_d_ = ((K x E_d_) – 1) / (K – 1), whereby K = number of sets, d = factor number (in this case only one factor was calculated), and E = the eigenvalue of the factor/index. rev = recoded in reverse order

### Trichotomisation

**Fig. 2:**
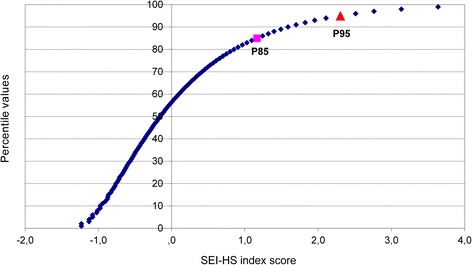
Distribution of SEI-HS scores. Each *dot* represents 1% of the weighted study population. The *pink square* marks the 85 percentile. The *red triangle* marks the 95 percentile

The 85th and 95th percentile scores of the index and dimension scales were calculated in the weighted total sample (Fig. 2). This resulted in corollary prevalence rates between 5.0 and 5.2% “moderate to strong” exclusion and between 8.6 and 11.8% “some” exclusion on the general index and the dimensions scales. Prevalence rates in the development and validation samples were very similar.

### Validation of the SEI-HS

#### Content validity

The data in Table 3 show that the SEI-HS items covered all the aspects of SE that form part of the SCP index. All four dimensions of SE were measured with three or more items. Only one item had a low component loading i.e. ‘didn’t receive medical or dental treatment’ (component loading 0.27); and one item had a low weight i.e. ‘I have enough money to heat my home’ (weight 0.09). The eigenvalues of the dimension scales ranged from 0.43 for Normative Integration tot 0.54 for Social Participation and Social Rights, which is largely consistent with the eigenvalues of the SCP dimension scales. As expected, the scores on the SEI-HS were right-skewed (Fig. 2) with mean 0 and standard deviation 1, i.e. similar to the SCP Index.

#### Internal consistency

The SEI-HS has a sufficient canonical correlation (0.33). This is somewhat lower than the correlation found for the SCP Index (0.38). Cronbach’s alpha for the dimension scales Social Participation and Material Deprivation were sufficient (α ≥ 0.70). The Social Rights and Normative Integration scales, however, had insufficient Cronbach’s alpha coefficients of respectively 0.34 and 0.30. The internal consistencies of the SEI-HS scale were all higher than those of the SCP dimension scales.

#### Internal structure

Table 4 presents the intercorrelations between the dimension scales and general index. As expected, the SEI-HS showed strong positive correlations between the scales and the general index (*r* > = 0.60) and weak positive correlations between the dimension scales interact (0.20 < = *r* < 0.40), which are comparable to those of the SCP Index. The results showed an acceptable model fit with all factor loadings significant at the 0.001 level 2; RMSEA =0.057 (HI90 = 0.057); TLI = 0.827; CFI = 0.872 and Hoelter’s .05 Index = 407.

**Table 4:** Pearson correlations between the subscales (dimensions^a^) and the general index, SEI-HS (development sample) and SCP index

Correlation between:	SEI-HS	SCP index^b^
General index x SP	0.73*	0.76
General index x MD	0.69*	0.70
General index x SR	0.72*	0.77
General index x NI	0.64*	0.68
SP x MD	0.34*	0.35
SP x SR	0.37*	0.43
SP x NI	0.31*	0.41
MD x SR	0.34*	0.44
MD x NI	0.26*	0.28
SR x NI	0.28*	0.34

*p < 0.001

^a^ *SP* social participation, *MD* material deprivation, *SR* social rights, *NI* normative integration

^b^Vrooman and Hoff [34]

#### Construct validity

As shown in Table 5, all construct validity hypotheses were confirmed at the .001 level of confidence. Poor labour market position and poor health (poor perceived health and high risk for anxiety and depression disorder) had the strongest relationships with the SEI-HS. Also the factors non-Western ethnic background, low income, living alone, low education, living in a deprived neighbourhood and single parenthood, were all associated with a higher level of SE. The associations were generally stronger with the SEI-HS than with the SCP index (Table 5). An exception was the factor ‘single parenthood’.

**Table 5:** Association between SEI-HS and known risk factors and correlates (development sample) and comparison with SCP index

		SEI-HS		SCP index^a^	
		(Development sample: *N* = 129.464)		(*N* = 574)	
		β^b^	*p*	β^b^	*p*
Educational level	Low educational level (no education and primary school)	0.18	***	0.12	**
Ethnic background	Non-Western ethnic background	0.27	***	0.18	**
Family situation	Single parent with underage child(ren)	0.07	***	0.13	**
	Living alone	0.19	***	0.16	**
Labour market position (64 years or younger)	Unemployed and/or recipient of social security or disability benefits. (SCP: Receives unemployment benefit, disability benefit or social assistance benefit)	0.31	***	−0.03	ns
	No paid job	0.21	***	0.02	ns
Income	Low income^c^ (SCP: Less than average household income)	0.26	***	0.23	**
Health	Self-rated health fair or poor	0.31	***	0.19	**
	Diagnosed with at least one chronic condition. (SCP: Suffers from a disability or a chronic condition)	0.13	***	0.09	*
	Severe functional limitations in mobility, vision or hearing	0.27	***		
	High risk for anxiety and depression disorder. (SCP: Low subjective well-being)^d^	0.36	***	0.30	**
Neighbourhood	Living in deprived neighbourhood	0.18	***		

Explanatory note. Linear regression analyses were used to assess relationships between SEI-HS and known risk factors and correlates. Construct validity was considered satisfactory if at least 75% of the associations were in correspondence with predefined hypotheses

* Significant effect, *p* < 0.05; ** Significant effect, *p* < 0.01; *** Significant effect, *p* < 0.001; ns Not significant, *p* > =0.05

^a^Vrooman and Hoff [34]

^b^Standardised regression coefficients

^c^Low income = lowest quintile standardised of yearly household income (2010) i.e. below 15.200 Euro. (Data obtained from Statistics Netherlands)

^d^Kessler psychological distress scale (K10), score 30 or higher

#### Generalisability

No marked differences in the canonical functions were found between the analysis in the development and validation samples. The eigenvalues of the index and subscale Social Participation were similar in the two samples. The eigenvalues of the subscales Material Deprivation, Social Rights and Normative Integration were almost similar: 0.50, 0.52 and 0.44 respectively in the validation as opposed to 0.49, 0.53 and 0.43 in the development sample. The same holds true for component loadings and weights.

## Discussion

The findings of this study show that we succeeded in developing a reliable and valid multidimensional measure for SE, the Social Exclusion Index for Health Surveys or SEI-HS. The OVERALS analyses empirically confirmed our multidimensional model with SE as the underlying latent construct. The limitations we encountered in previous retrospective research with regard to content validity and generalisability were successfully tackled in this nationwide prospective study. Content validity was enhanced by the addition of extra items. Instead of three dimensions in our previous study, the SEI-HS measured all four dimensions of SE. Generalisability was enhanced by successful replication of the SEI-HS in a representative validation sample. Other psychometric properties were found to be satisfactory to good and in line with the original SCP Index. Low to moderate intercorrelations between index and subscales confirmed the internal structure of the SEI-HS and construct validity was established through hypothesis testing.

The internal consistencies of two of the SEI-HS dimension scales were found to be weak. Both the Social Rights and Normative Integration scales had Cronbach’s alpha coefficients lower than 0.70. By using canonical correlation analysis to construct a measure for SE, we selected those elements from the underlying theoretical dimensions that interrelate with one another and form a coherent construct. Access to basic social rights and normative integration, but also social participation and material deprivation are broader concepts than the dimension scales resulting from these analyses. Access to basic social rights, for example, also comprises e.g. access to other public and private services such as education, legal aid, acceptance for insurance and banking and help with finding a job. When empirically tested, these forms of access proved not relevant to the concept of SE, at least not in the general population in the Netherlands [33, 34]. These aspects of basic social rights were therefore not included in the Social Rights scale. The SEI-HS dimension scales are thus relevant and of value only in the context of the concept SE.

One of the study’s strengths is the use of a sound and validated instrument to supplement items on domains where the Netherlands PHM fell short. The SCP items were originally selected by the SCP with nonlinear canonical correlation analysis from an item pool of 232 items derived from extensive literature and empirical research, focus groups and cognitive tests [5, 33, 34, 50]. Thus, the selected items not only have a strong theoretically basis, but also a strong empirical basis. The findings of this study supported our choice. The SCP items perfectly complemented the existing PHM items. Together, they covered the full width of the theoretical construct and produced an empirically sound and valid instrument.

Another strong point is the study’s large and representative sample. Over half a million adults were invited to participate in this study and data from over 250.000 respondents were available for analysis. The widespread participation allowed us to extend the generalisability of the SEI-HS to the whole Dutch adult population and calculate national reference data, by sex, age group, urbanicity, ethnical background and educational level; thus providing a benchmark for Community Health Services and municipalities to compare their local data with [51]^3^. The high number of Community Health Services that took part in this study not only advanced the quality of the research, it also indicates the pertinence of SE to the field of public health in the Netherlands. The fact that 19 out of 28 Dutch Community Health Services (covering over 70% of the Dutch population) made space available in their surveys for additional SE items is illustrative of the importance given to SE. Most Community Health Services have since published local figures and reports on SE, with local policy recommendations (e.g. [52–57]). This provides a good demonstration of the value and potential of a SE measure for the public health sector.

The response rate of this study was 45.7%, which is typical for population surveys in the Netherlands [58, 59]. The Dutch PHM employs a systematic strategy to minimise non-response error. The strategy includes measures to increase the general response rate such as pre-survey notification and media coverage in e.g. local newspapers and social media, a mixed mode approach combining web and paper questionnaires, multiple reminders and specific measures to increase representation of hard to reach groups e.g. home visits, translated questionnaires, assistance in completing the questionnaire and oversampling. Lastly, it includes robust weighting procedures to reduce non-response error. We believe that sample representativity is sufficiently guaranteed by the taken measures, particularly for our purpose, the estimation of the parameters of the SEI-HS measure. Although additional analyses (not shown) indicate that the level of SE in the study population has relatively limited effect on the parameters of the SEI-HS, we recommend to retest the SEI-HS in different samples with full inclusion of population groups that are particularly vulnerable to SE. As is common practice in population health surveillance, only persons living in private households were included into the Dutch PHM, thereby excluding groups such as homeless persons and detainees. In the Netherlands, 0.2% of the adult population was estimated in 2012 as being homeless and 1.6% lived in an institutional household, mostly elderly persons [CBS Statline]. Prevalence rates should therefore be interpreted with caution.

The index and scale scores were trichotomised using 85th and 95th percentile scores, resulting in three categories of SE: “moderate to strong” exclusion (score > P95), “some” exclusion (P85 < score ≤ P95) and “little or no” exclusion (score ≤ P85). There are a number of reasons for selecting P85 and P95 as cut-off points. Firstly, using these cut-off points enhances the applicability of the instrument in public health policy. Municipalities prefer to target comprehensive (and costly) interventions at well-defined small population groups with the highest risk, while more general preventive policies may focus on wider population groups. 5% and 10%, respectively, are considered here as useful guidelines. Secondly, the categorisation fits the right-skewed distribution of the index scores, indicating that the largest part of the population is not excluded (Fig. 2). Lastly, the choice of the two cut-off points does justice to the relative and continuous character of SE. It allows for the possibility of social groups being differentially included rather than suggesting an artificial dichotomy between included and excluded groups and avoids the stigma of labelling particular groups [7]. Despite this substantiation, the choice of P85 and P95 as cut-off points remains arbitrary. A certain degree of arbitrariness is inevitable in a continuous phenomenon such as SE, where there is no set point at which a person is or is not excluded. Using objective methods such as ROC curves for determining cut-off points would only disguise the inherent arbitrariness.

Although the SEI-HS was designed specifically for inclusion in the Netherlands PHM, it is highly suitable for application in public health surveys in countries with similar physical, economic and social conditions where it complements the current validated SE measures. Because of its potential for calculating composite scores and the absence of health as a constituent part of the index, the SEI-HS. allows researchers to study the relationship between SE and health, knowledge indispensable for designing effective policies to diminish socioeconomic health inequalities. This is a promising development as SE provides a broader and thereby potentially more effective range of policy options than concepts like poverty and loneliness [3, 60, 61]. The SEI-HS can be used in identifying risk groups for targeting specific interventions and monitoring their impact over time [6, 7, 61], and in raising the profile and visibility of excluded groups and alerting professionals to the diverse causes and consequences of SE [13]. Finally, our approach to the development of a short embedded index with canonical correlation analyses, may serve as an example to the further development of key public health measures.

## Conclusions

We have described the development of an instrument to measure the multidimensional concept SE and its validation in a major national public health survey. All four dimensions of SE could be measured and overall, the SEI-HS showed satisfactory to good psychometric properties. The SEI-HS enables researchers to take a next step in the advancement of much needed knowledge on SE and health. The study also provides valuable insights in how to develop embedded measures for public health surveillance.

## Additional files

Additional file 1: Category quantifications SEI-HS items dimension (limited) Social Participation. The figure shows for each item of the dimension Social Participation the relationship between the original category and the quantification resulting from the canonical correlation analysis. Categories indicating little or no social exclusion received the lowest quantifications and categories indicating high levels of social exclusion received the highest values. The category quantifications were used to calculate the Social Participation scale score by multiplying them with their item weights (Table 3); and adding up the results. (TIF 2369 kb)
Additional file 2: Category quantifications SEI-HS items dimension (inadequate access to basic) Social Rights. The figure shows for each item of the dimension Social Rights the relationship between the original category and the quantification resulting from the canonical correlation analysis. Categories indicating little or no social exclusion received the lowest quantifications and categories indicating high levels of social exclusion received the highest values. The category quantifications were used to calculate the Social Rights scale score by multiplying them with their item weights (Table 3); and adding up the results. (TIF 1548 kb)
Additional file 3: Category quantifications SEI-HS items dimension (lack of) Normative Integration. The figure shows for each item of the dimension BI the relationship between the original category and the quantification resulting from the canonical correlation analysis. Categories indicating little or no social exclusion received the lowest quantifications and categories indicating high levels of social exclusion received the highest values. The category quantifications were used to calculate the Normative Integration scale score by multiplying them with their item weights (Table 3); and adding up the results. (TIF 1401 kb)

## Abbreviations

CBS: Statistics Netherlands
PHM: Public Health Monitor
rev: Recoded in reverse order
SCP: Netherlands Institute for Social Research
SE: Social exclusion
SEI-HS: Social Exclusion Index-for Health Surveys

## Footnotes

1 The default setting of OVERALS removes records with missing values on all four dimensions i.e. 4 out of 17 items. Because of the diversity in items we reduced the maximum to 3 missing values.

2 The factor loadings in the dimensions Social Participation ranged from 0.26 to 0.77; Material Deprivation from 0.52 to 0.59; Social Rights from 0.23 to 0.44; and Normative Integration from 0.30 to 0.41.

3 The syntax to calculate SEI-HS index and scale scores are available from the corresponding author.
