# Supplementary figures and images for: Social Exclusion Index-for Health Surveys (SEI-HS): a prospective nationwide study to extend and validate a multidimensional social exclusion questionnaire
Source: BMC Public Health. 2017 Mar 14;17:253. doi: 10.1186/s12889-017-4175-1 (PMC5348771; doi:10.1186/s12889-017-4175-1)

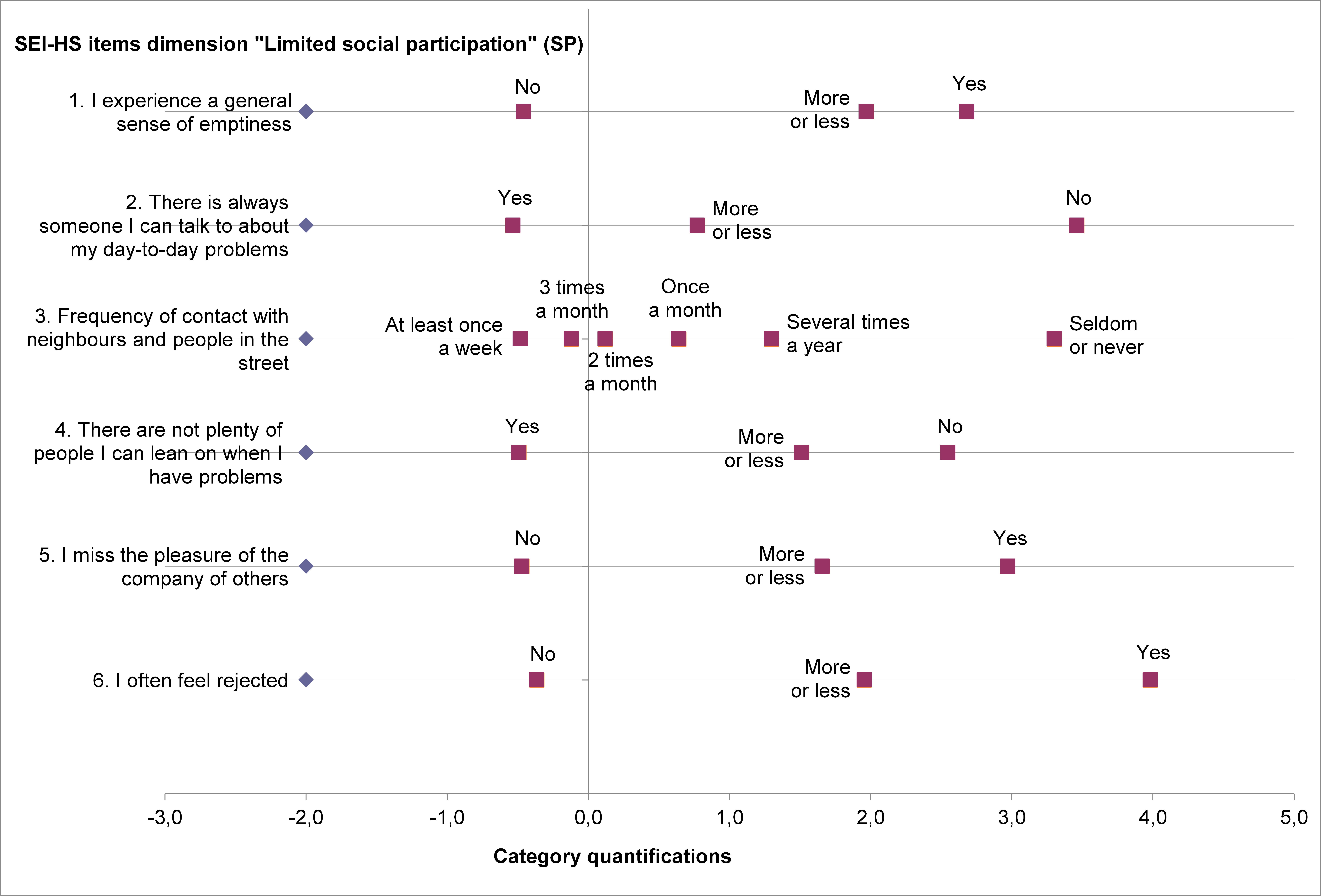

Supplement: Additional file 1: — Category quantifications SEI-HS items dimension (limited) Social Participation. The figure shows for each item of the dimension Social Participation the relationship between the original category and the quantification resulting from the canonical correlation analysis. Categories indicating little or no social exclusion received the lowest quantifications and categories indicating high levels of social exclusion received the highest values. The category quantifications were used to calculate the Social Participation scale score by multiplying them with their item weights (Table 3); and adding up the results. (TIF 2369 kb) [file 12889_2017_4175_MOESM1_ESM.tif]

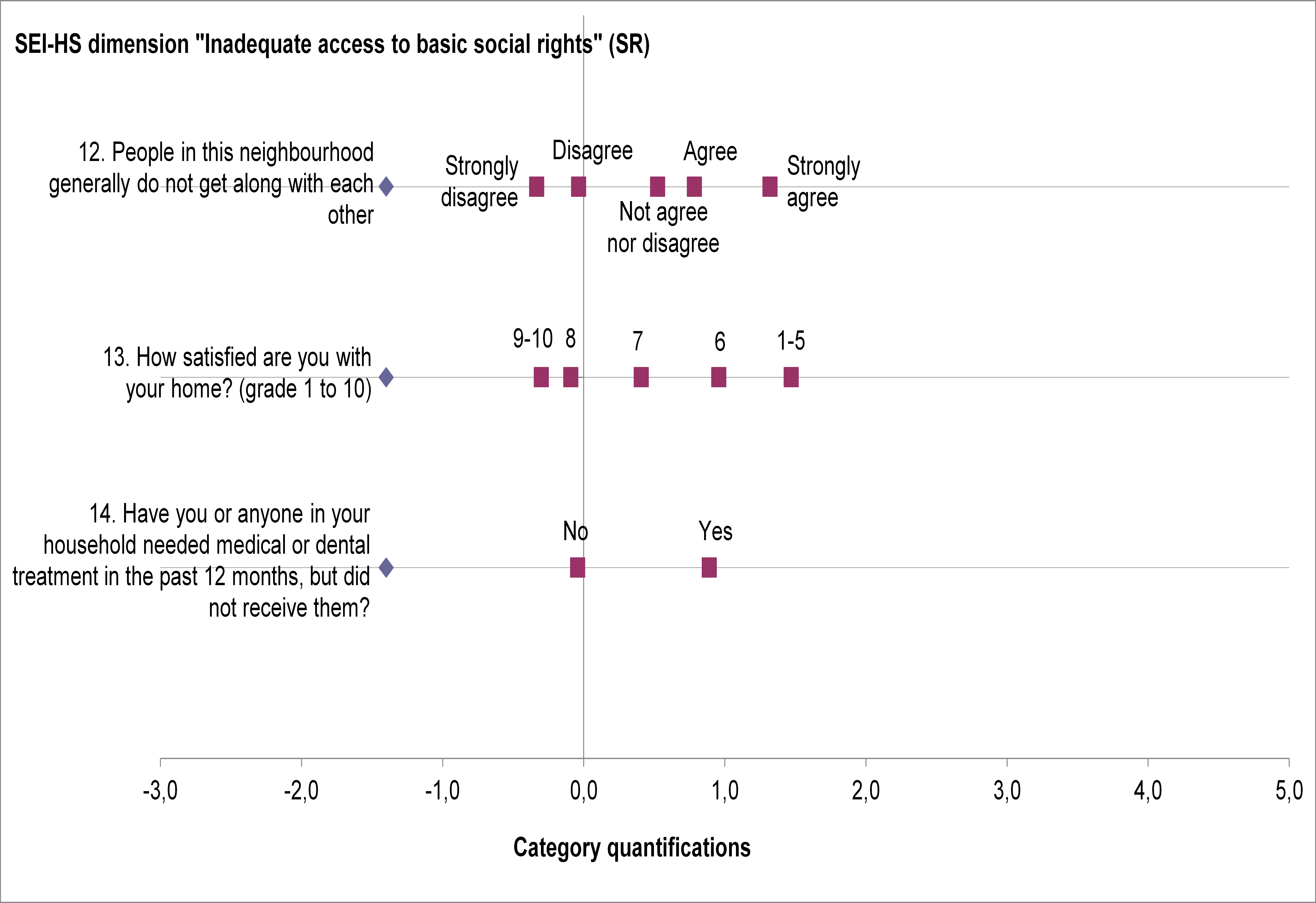

Supplement: Additional file 2: — Category quantifications SEI-HS items dimension (inadequate access to basic) Social Rights. The figure shows for each item of the dimension Social Rights the relationship between the original category and the quantification resulting from the canonical correlation analysis. Categories indicating little or no social exclusion received the lowest quantifications and categories indicating high levels of social exclusion received the highest values. The category quantifications were used to calculate the Social Rights scale score by multiplying them with their item weights (Table 3); and adding up the results. (TIF 1548 kb) [file 12889_2017_4175_MOESM2_ESM.tif]

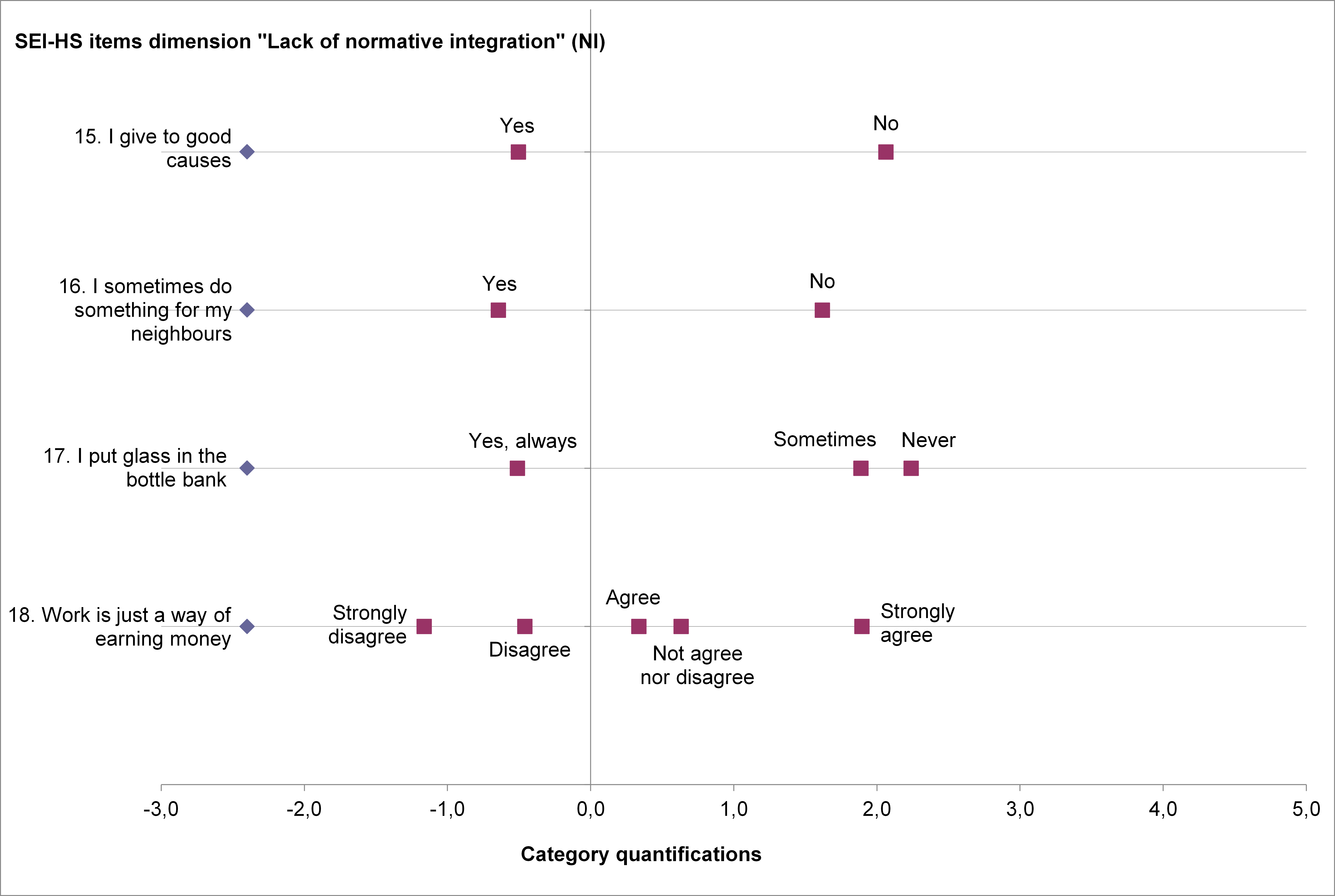

Supplement: Additional file 3: — Category quantifications SEI-HS items dimension (lack of) Normative Integration. The figure shows for each item of the dimension BI the relationship between the original category and the quantification resulting from the canonical correlation analysis. Categories indicating little or no social exclusion received the lowest quantifications and categories indicating high levels of social exclusion received the highest values. The category quantifications were used to calculate the Normative Integration scale score by multiplying them with their item weights (Table 3); and adding up the results. (TIF 1401 kb) [file 12889_2017_4175_MOESM3_ESM.tif]
